# Glucagon-Like Peptide-2 Analogue ZP1849 Augments Colonic Anastomotic Wound Healing

**DOI:** 10.1155/2020/8460508

**Published:** 2020-10-09

**Authors:** Marie Kjaer, Wayne Russell, Peter Schjerling, Elena Cottarelli, Kennet N. Christjansen, Ditte M. G. Olsen, Peter-Martin Krarup, Lene Jessen, Mark Berner-Hansen, Lars N. Jorgensen, Magnus S. Ågren

**Affiliations:** ^1^Digestive Disease Center, Bispebjerg Hospital, University of Copenhagen, 2400 Copenhagen NV, Denmark; ^2^Zealand Pharma A/S, 2600 Glostrup, Denmark; ^3^Institute of Sports Medicine Copenhagen and Department of Biomedical Sciences, Bispebjerg Hospital, University of Copenhagen, 2400 Copenhagen NV, Denmark; ^4^Copenhagen Wound Healing Center, Bispebjerg Hospital, University of Copenhagen, 2400 Copenhagen NV, Denmark

## Abstract

**Background:**

The enteroendocrine hormone glucagon-like peptide- (GLP-) 2 is a potent trophic factor in the gastrointestinal tract. The GLP-2 receptor (GLP-2R) is expressed in the stroma of the large bowel wall, which is the major therapeutic target area to prevent anastomotic leakage. We investigated the efficacy of the long-acting GLP-2 analogue ZP1849 on colonic anastomotic wound healing.

**Methods:**

Eighty-seven male Wistar rats were stratified into four groups and received daily treatment with vehicle or ZP1849 starting one day before (day -1) end-to-end anastomosis was constructed in the left colon on day 0, and on days 0 (resected colon segment), 3, and 5, gene expressions of GLP-2R, Ki67, insulin-like growth factor- (IGF-) 1, type I (COL1A1) and type III (COL3A1) procollagens, cyclooxygenase- (COX-) 1, COX-2, and matrix metalloproteinase- (MMP-) 7 were quantified by RT-qPCR. Breaking strength, myeloperoxidase (MPO), transforming growth factor- (TGF-) *β*1, and soluble collagen proteins were measured on days 3 and 5.

**Results:**

ZP1849 treatment increased Ki67 (*P* < 0.0001) and IGF-1 (*P* < 0.05) mRNA levels in noninjured colon day 0, and postoperatively in the anastomotic wounds compared to vehicle-treated rats. ZP1849-treated rats had increased (*P* = 0.042) anastomotic breaking strength at day 5 compared with vehicle. COL1A1 and COL3A1 mRNA levels (*P* < 0.0001) and soluble collagen proteins (*P* < 0.05) increased from day 3 to day 5 in ZP1849-treated rats, but not in vehicle-treated rats. COX-2 mRNA and MPO protein levels decreased from day 3 to day 5 (*P* < 0.001) in both groups. ZP1849 treatment reduced TGF-*β*1 protein levels on day 5 (*P* < 0.001) but did not impact MMP-7 transcription.

**Conclusions:**

The GLP-2 analogue ZP1849 increased breaking strength, IGF-1 expression, and cell proliferation, which may be beneficial for colonic anastomotic wound healing.

## 1. Introduction

Anastomotic leakage (AL) after colorectal surgery is associated with increased morbidity, increased cancer recurrence, and reduced long-term survival [1–3]. There is a high unmet medical need for pharmacological interventions to prevent or mitigate AL [4, 5].

The extracellular matrix in the colon is paramount for anastomosis repair. The predominant collagen subtypes in colorectal tissue are type I and type III. Recently, we showed a decreased collagen synthesis capacity, measured as type I procollagen (COL1A1) and type III procollagen (COL3A1) mRNA levels, the more distal the anastomosis in colorectal patients [6]. This alteration in the collagen synthesis capacity is then a possible explanation for the higher incidence of AL the lower the anastomosis.

Several growth factors have been investigated in experimental models to enhance anastomotic wound healing but with mediocre results [5, 7]. Glucagon-like peptide- (GLP-) 2 is a potent enteroendocrine hormone with numerous beneficial effects in the setting of experimental intestinal injury [8]. Unlike most growth factors, GLP-2 acts exclusively on connective tissue cells expressing its receptor (GLP-2R) in the gastrointestinal tract [9]. This property makes the GLP-2 pathway an attractive target for therapeutic intervention.

Activation of GLP-2R increases the production and secretion of several growth factors [10]. Insulin-like growth factor- (IGF-) 1 is an important mediator of the trophic effects of GLP-2. IGF-1 treatment increases collagen deposition and the biomechanical strength of colonic anastomoses [11, 12].

To our knowledge, only a few studies on GLP-2 in intestinal anastomotic healing have been reported [13, 14], and GLP-2 as a pharmacological intervention has not been fully explored. In a previous study, the effect of GLP-2 treatment was assessed by the bursting pressure of the anastomosis [13]. Notably, we found that breaking strength, another metric to assess anastomotic wound healing, is the more valid method to evaluate early anastomotic wound healing (see Supplementary Materials (available here)). We therefore investigated the effect of a long-acting GLP-2 analogue (ZP1849; Zealand Pharma A/S) on colonic anastomotic wound healing in a rat model with anastomotic breaking strength as the primary outcome and molecular markers as secondary outcomes. We hypothesized that GLP-2 would improve anastomotic wound healing by promoting the local IGF-1 expression, resulting in increased collagen synthesis measured by COL1A1 and COL3A1 mRNA and soluble collagen proteins and increased anastomotic strength. Furthermore, we studied the effect of ZP1849 treatment on key genes in anastomotic wound healing associated with proliferation (Ki67), inflammation (cyclooxygenase- (COX-) 1 and COX-2), and epithelialization (matrix metalloproteinase- (MMP-) 7).

## 2. Materials and Methods

The study was conducted at Zealand Pharma A/S over a 6-week period and carried out in accordance with guidelines from the Ministry of Environment and Food of Denmark and in accordance with the institutional license (2016-15-0201-01037). The ARRIVE guidelines were followed.

### 2.1. Study Drug

Endogenous GLP-2 is composed of 33 amino acids and has a short half-life of about 7 minutes due to proteolytic cleavage by dipeptidyl peptidase IV [15]. Chemical substitution of the amino acid alanine to glutamine at position 2 extends the half-life of GLP-2, and this molecule (teduglutide) is approved for the treatment of short bowel syndrome [16]. ZP1849 is a 39 amino acid peptide that differs from endogenous GLP-2 by comprising the position 2 modification and 7 additional amino acid substitutions, and a C-terminal tail consisting of 6 lysines. ZP1849 is as such a long-acting GLP-2 analogue with extended pharmacokinetic half-life properties [17].

### 2.2. Animals

Eighty-seven male Wistar albino rats (Charles River Laboratories, Sulzfeld, Germany) weighing 215-285 g were used. Two rats were housed per standard type IV cage with sawdust bedding. Animals were kept in a room at 20–22°C, 50–80% relative humidity, and with lights on from 6 AM to 6 PM. The rats had free access to tap water acidified with citric acid to pH 3.6 and regular chow (Altromin 1324). The animals were acclimatized for at least seven days before mock treatment and surgery. The rats received mock treatment daily at days -4, -3, -2, and -1 consisting of 1 mL s.c. 0.9% NaCl (saline), a postoperative nutrient water gel (ClearH_2_O®) provided in the cage, and handling by the caretakers. On days 0, 1, 2, and 3, the animals had free access to DietGel®Recovery gels in the cage.

### 2.3. Experimental Design

Rats were stratified to four groups according to body weight: vehicle treatment, termination day 3 (n=23); ZP1849 treatment, termination day 3 (n=22); vehicle treatment, termination day 5 (n=21); ZP1849 treatment, termination day 5 (n=21). Treatment (5 mL/kg s.c.) with vehicle phosphate-buffered saline (PBS) or ZP1849 (225 nmol/kg in PBS) started one day before anastomosis surgery (day -1). Animals were injected once daily in the afternoon. On day 0, end-to-end colonic anastomoses were constructed, and the resected tissue was saved for biochemical analyses. On days 3 and 5, venous blood was collected from the tail into EDTA-coated tubes, the animals were killed, and breaking strength was determined, and anastomotic tissue procured for biochemical analyses.

### 2.4. Anaesthesia, Surgical Procedure, Biomechanical Testing, and Tissue Procurement

Operations, anastomotic evaluations, biomechanical testing, and tissue preparations were performed by one author (M. K.).

On day 0, buprenorphine (0.03 mg/kg s.c.; Temgesic®) was given preoperatively. Anaesthesia was introduced with isoflurane (IsoFlo® Vet) at 4.0%/O_2_ (1.5 L/minute) and maintained at 2.0-2.5%/O_2_. The animals were placed on a heating pad at 37°C. Bupivacaine (2.0 mg/kg s.c.; Marcaine®) was administered to the incision site. A 40 mm midline incision was made under aseptic conditions, a 10 mm segment of the left colon was resected 60 mm from the anus, and faecal content was manually removed. The noninjured colon segment was snap-frozen in liquid nitrogen and stored at -80°C until analysed for mRNAs. An end-to-end single-layer anastomosis was made using 8 interrupted, inverted polypropylene monofilament 6/0 sutures (Prolene®) placed 2 mm from the resection margins [18, 19]. Saline (37°C) was applied to the abdominal cavity (5 mL) and subcutaneous tissue (2.5 mL). The abdominal muscles and the transverse fascia were closed with interrupted 4/0 sutures (Ethilon™II). The skin was closed using 7-8 titanium clips (Reflex® 9 mm). Lidocaine 2.5% and prilocaine 2.5% cream (EMLA®) was applied to the closed incision. Buprenorphine (0.03 mg/kg s.c.) was provided in 8-hour intervals on day 0 and twice daily on days 1 and 2, and additional doses were given when required. Saline (2.0 mL s.c.) was injected on day 1, and meloxicam (2 mg/kg s.c.; Metacam®) was given once daily in the afternoon days 1, 2, 3, and 4.

On days 3 or 5, rats were anesthetized with N_2_O/isoflurane. The anastomosis was freed of adhesions, resected in toto, transported to Petri dish with saline, and evaluated macroscopically for AL [18]. The animals were killed by cervical dislocation. The anastomosis was then subjected to biomechanical testing (see Figure S2 in the Supplementary Materials for details), and the breaking strength was determined [20]. The disrupted anastomotic wound was bisected longitudinally into two biopsies, snap-frozen in cryogenic tubes immersed in liquid nitrogen, and stored at -80°C. One biopsy was used for MPO and TGF-*β*1 analyses. The other biopsy was cut into two pieces longitudinally at -20°C; one piece (<50 mg) was used for the mRNA analyses and the other piece for soluble collagen analyses.

### 2.5. ZP1849 Plasma Level Determination

Plasma was separated by centrifugation at 8,300 x g for 5 minutes at 4°C and stored at -80°C. The ZP1849 concentration (5-1000 nM) was determined with an added internal standard (analogue structurally like ZP1849). Following solid-phase extraction using Oasis MAX *μ*Elution plates (Waters, Milford, MA, USA), samples were diluted and analysed using liquid chromatography-tandem mass spectrometry (Acquity UPLC system, Xevo TQ-S, and MassLynx v4.0 software, Waters).

### 2.6. mRNA Analyses by Reverse Transcription Quantitative Polymerase Chain Reaction (RT-qPCR)

The resected noninjured colon and the anastomotic tissue were cut into thin slices at -20°C and transferred to homogenization tubes containing five stainless steel beads of 2.3 mm in diameter (BioSpec Products, Bartlesville, OK, USA) and homogenized in 1000 *μ*L TRI-reagent® [21]. Samples were mechanically disrupted using a FastPrep®-24 instrument at speed level 4 for 15 seconds and cooled on ice. This procedure was repeated three times. Subsequently, 100 *μ*L bromochloropropane was added to separate the extract into an aqueous and an organic phase. Following isolation of the aqueous phase, RNA was purified using the Direct-zol™ RNA MicroPrep Kit. The RNA was eluted in 30 *μ*L RNase-free water. RNA concentration and purity were determined by spectroscopy at 260, 280, 240, and 320 nm, and RNA quality was confirmed by agarose gel electrophoresis.

Total RNA (500 ng) was converted to complementary DNA (cDNA) in 20 *μ*L reaction buffer using Omniscript reverse transcriptase. For each target mRNA, 5 *μ*L of 20x diluted cDNA (in 10 mM Tris, 1 mM EDTA buffer, pH 8.0 with 1 ng/*μ*L salmon DNA) was amplified in a 25 *μ*L SYBR Green polymerase chain reaction (PCR) containing 1x QuantiTect SYBR Green Master Mix and 100 nM of each primer (Table 1) on a real-time PCR machine (MX3005P; Stratagene, La Jolla, CA, USA). The threshold cycle values were related to a standard curve made with cloned PCR products or DNA Ultramer oligonucleotides (Integrated DNA Technologies, Leuven, Belgium) to determine the relative difference between unknown samples, accounting for the PCR efficiency. The specificity of the PCR products was confirmed by melting curve analysis after amplification. The large ribosomal protein P0 (RPLP0) mRNA was chosen as an internal control for normalization, as RPLP0 mRNA has been suggested to be constitutively expressed [22]. Glyceraldehyde 3-phosphate dehydrogenase (GAPDH) is another common mRNA control and was measured to test RPLP0 mRNA stability. The ratio of GAPDH/RPLP0 appeared to be stable (no significant difference between groups), and RPLP0 was chosen for normalization.

### 2.7. Protein Analyses of the Anastomoses

Anastomotic tissue was homogenized in 1.5 mL PBS containing a proteinase inhibitor cocktail and 1 mM EDTA (pH 7.4) in tubes with stainless steel beads (Precellys® MK28 Lysing Kit). The tissue was disintegrated three times for 10 seconds at 6,500 rpm with 20-second breaks using the Precellys® 24 homogenizer. The homogenate was centrifuged at 20,000 x g for 30 minutes at 4°C, and the supernatant was stored at -80°C. MPO (EKR284; Nordic BioSite, Täby, Sweden) and TGF-*β*1 (ab119558; Abcam, Cambridge, UK) were analysed by rat ELISA kits. Soluble collagens were eluted from the tissue using a buffer (pH 6.0) containing 1 M NaCl for 24 hours at 4°C and determined by the Sircol assay [19]. MPO, TGF-*β*1, and soluble collagen were normalized to total protein determined by the Lowry assay.

### 2.8. Statistical Analyses

The sample size calculation was based on literature data from the same animal model: the mean ± standard deviation (SD) of anastomotic breaking strength was 1.19 ± 0.34 N in the control group compared with 1.54 ± 0.40 N in the intervention group on postoperative day 3 [23]. Based on a significance level of 5% and 80% power, it would require 18 animals in each group to detect a significant difference in breaking strength from 1.19 N to 1.54 N (average SD: 0.37 N). Therefore, we decided to include 22 animals per group to account for mortality.

mRNA targets in the noninjured resected colon and anastomotic wounds were normalized to RPLP0, expressed as fold change relative to the noninjured colon from vehicle-treated control rats, and log-transformed before statistical analyses. mRNA levels in the noninjured colon were analysed by unpaired Student's *t*-test. A paired Student's *t*-test was used to assess the change in mRNA targets from day 0 to day 3. To assess the effect of treatment (vehicle and ZP1849) and time (day 3 and day 5), a two-way factorial ANOVA was applied, and in case of a statistically significant interaction (treatment × time), this was followed by a Tukey's honestly significant difference post hoc test. Statistical analyses were two-sided and performed using SPSS Statistics 25.0 software (IBM). The level of statistical significance was set to *P* < 0.05.

## 3. Results

Four groups of animals were used. Two parallel groups received vehicle and ZP1849 and were killed on day 3 for anastomotic breaking strength measurements and biochemical analyses. The other two parallel vehicle and ZP1849 groups were killed on day 5 also for anastomotic breaking strength measurements and biochemical analyses. Treatment with vehicle or ZP1849 started one day before the anastomoses were constructed (day -1).

Two ZP1849-treated rats from the day-5 group were euthanized on day 1 due to poor health. There was no significant difference in body weight between the groups on the day of surgery. The body weights decreased postoperatively and were the lowest on day 3 (drop ~5%). Thereafter, the animals gained body weight at a similar rate for the two groups.

The ZP1849 plasma concentrations 15-18 hours after the last administration of ZP1849 ranged from 4.2 to 18.5 nmol/L. The mean ± SD concentrations were 11.5 ± 4.4 nmol/L on day 3 and 7.2 ± 2.7 nmol/L on day 5 in the ZP1849-treated groups. ZP1849 was undetectable in plasma in the vehicle groups.

### 3.1. Effects of GLP-2 Treatment on Gene Expression in Day-0 Noninjured Colon

**Figure 1:**
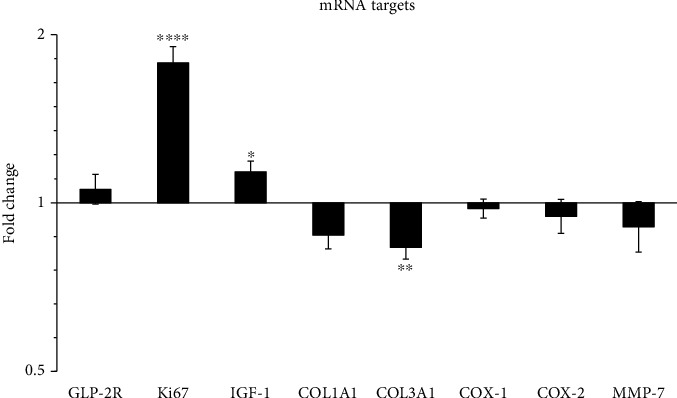
The effect of one-day ZP1849 treatment on the gene expression in the noninjured colon on day 0. The mRNA levels were normalized to RPLP0, shown as fold changes relative to vehicle-treated rats and tested as ZP1849 versus vehicle. ^∗^*P* < 0.05, ^∗∗^*P* < 0.01, and ^∗∗∗∗^*P* < 0.0001. Geometric mean ± back‐transformed SEM.

The effect of ZP1849 treatment for one day on the expression of the target genes was studied in the resected noninjured colon (Figure 1). ZP1849 treatment increased the Ki67 and IGF-1 mRNA levels and reduced the COL3A1 mRNA levels compared with vehicle treatment. ZP1849 treatment had no significant effects on the GLP-2R, COL1A1, COX-1, COX-2, or MMP-7 mRNA levels.

### 3.2. AL and Biomechanical Outcomes

**Figure 2:**
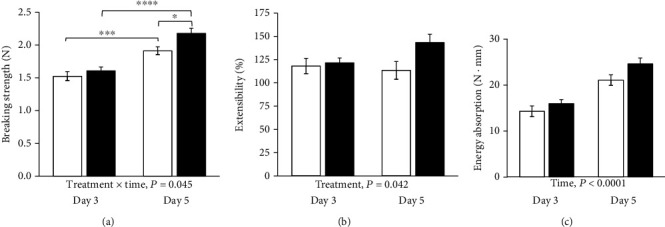
Anastomotic breaking strength (a), extensibility (b), and energy absorption (c). Open bars: vehicle; filled bars: ZP1849. ^∗^*P* < 0.05, ^∗∗∗^*P* < 0.001, and ^∗∗∗∗^*P* < 0.0001. Mean ± SEM.

No AL was observed. One day-3 anastomosis and one day-5 anastomosis in the placebo groups were damaged during preparation and were excluded from the breaking strength measurements. All anastomoses broke in the anastomotic line. Breaking strength increased from day 3 to day 5 in the vehicle-treated rats and in the ZP1849-treated rats. On day 5, the mean anastomotic breaking strength was 13% higher (*P* = 0.042) with ZP1849 treatment compared with vehicle (Figure 2(a)). The extensibility of the anastomoses was higher with ZP1849 on day 5 (Figure 2(b)). Energy absorption of the anastomoses increased from day 3 to day 5 with both treatments (Figure 2(c)).

### 3.3. Effects of GLP-2 Treatment on Anastomotic Gene Expression and MPO, TGF-*β*1, and Soluble Collagen Proteins in Anastomotic Wounds on Days 3 and 5

**Figure 3:**
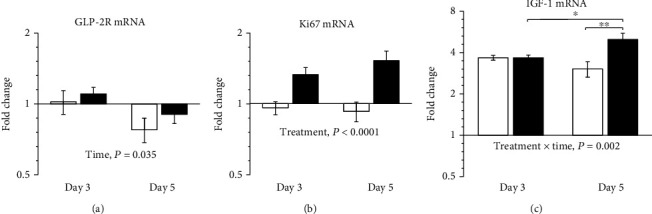
GLP-2R (a), Ki67 (b), and IGF-1 (c) mRNA levels in anastomotic wounds on days 3 and 5 normalized to RPLP0 and shown as fold changes relative to the noninjured colon of vehicle-treated rats on day 0. Open bars: vehicle; filled bars: ZP1849. ^∗^*P* < 0.05 and ^∗∗^*P* < 0.01. Geometric mean ± back‐transformed SEM.

The 8 selected mRNA targets were detected in all samples (Table 1). The gene expressions of IGF-1 (*P* < 0.0001), COL1A1 (*P* < 0.0001), COL3A1 (*P* < 0.0001), COX-2 (*P* = 0.005), and MMP-7 (*P* < 0.0001) were upregulated in the day-3 anastomotic wounds compared to the noninjured colon of vehicle-treated animals. The overall GLP-2R gene expression decreased from day 3 to day 5 with no effect of treatment (Figure 3(a)). Ki67 mRNA levels were higher in ZP1849-treated animals on days 3 and 5 compared with vehicle treatment (Figure 3(b)). IGF-1 mRNA levels increased from day 3 to day 5 in ZP1849-treated rats and were higher on day 5 compared with vehicle-treated rats (Figure 3(c)).

**Figure 4:**
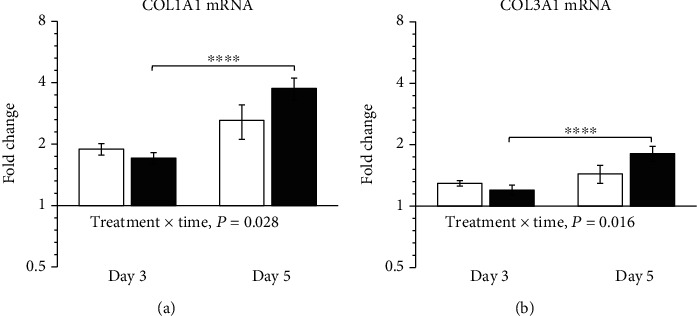
COL1A1 (a) and COL3A1 (b) mRNA levels in anastomotic wounds on days 3 and 5 were normalized to RPLP0 and shown as fold changes relative to the resected noninjured colon of vehicle-treated rats on day 0. Open bars: vehicle; filled bars: ZP1849. ^∗∗∗∗^*P* < 0.0001. Geometric mean ± back‐transformed SEM.

COL1A1 and COL3A1 mRNA levels increased from day 3 to day 5 in ZP1849-treated rats but not in vehicle-treated rats (Figure 4).

**Figure 5:**
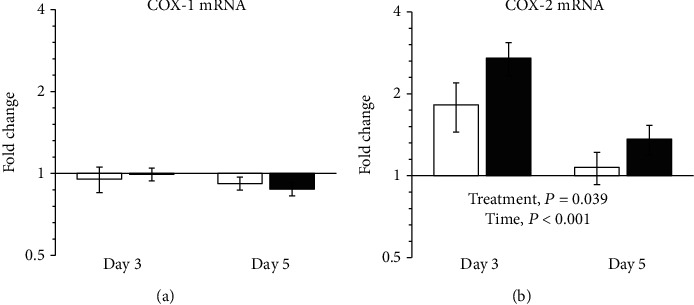
COX-1 (a) and COX-2 (b) mRNA levels in anastomotic wounds on days 3 and 5 were normalized to RPLP0 and are shown as fold changes relative to the noninjured colon of vehicle-treated rats on day 0. Open bars: vehicle; filled bars: ZP1849. Geometric mean ± back‐transformed SEM.

The gene expression of COX-1 did not change over time or with treatment (Figure 5(a)). COX-2 mRNA levels decreased with time. ZP1849 treatment increased COX-2 mRNA levels compared with vehicle treatment days 3 and 5 (Figure 5(b)).

MMP-7 mRNA levels increased (*P* < 0.0001) 21-fold to 24-fold in the anastomotic wounds compared to the noninjured colon in the four groups. There was no effect of time or treatment on the MMP-7 transcript levels.

MPO levels decreased from day 3 to day 5 with no significant difference between the ZP1849 and vehicle groups on day 3 or 5 (Table 2). On day 5, TGF-*β*1 levels were lower in the anastomotic wounds of ZP1849-treated animals compared with vehicle treatment (Table 2).

**Figure 6:**
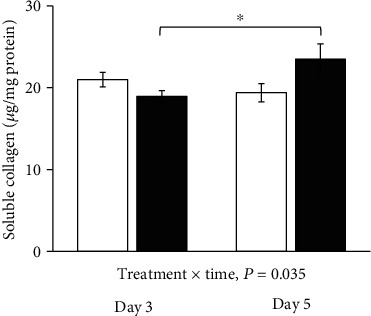
Soluble collagen protein levels in anastomotic wounds on days 3 and 5. Open bars: vehicle; filled bars: ZP1849. ^∗^*P* < 0.05. Mean ± SEM.

Soluble collagen protein levels increased from day 3 to day 5 in ZP1849-treated rats, but not in vehicle-treated rats (Figure 6). The soluble collagen levels did not differ between the vehicle and ZP1849 groups on the individual days 3 and 5.

## 4. Discussion

We have investigated the intestinotrophic effects of the long-acting GLP-2 analogue ZP1849 on the healing of colon anastomoses in an animal model. The main findings were that ZP1849 treatment increased the biomechanical strength of the colonic anastomoses and increased cell proliferation, indicated by the elevated Ki67 expression, and local IGF-1 gene expression.

Our results on breaking strength deviate from the study by Redstone et al. who were unable to demonstrate augmented anastomotic strength after GLP-2 treatment using another biomechanical metric (bursting pressure) on postoperative day 5 [13]. This lack of effect on anastomotic strength may be explained using different biomechanical metrics, the use of other GLP-2 analogues, or the differences in sample size. Further experiments are needed to investigate this in more depth.

We could reproduce the results of de Oliveira et al. [24] where increased IGF-1 mRNA levels were observed in the anastomotic wounds compared with the noninjured colon, emphasizing the importance of IGF-1 in anastomotic wound healing. ZP1849 treatment stimulated an early increase in IGF-1 expression in the noninjured colon, and IGF-1 mRNA levels were further increased in the anastomotic wounds on day 5 compared to vehicle.

Collagen is responsible for the biomechanical strength of anastomoses [25]. ZP1849 downregulated COL3A1 in the noninjured colon, which is in keeping with previous findings with other GLP-2 analogues [13]. The gene expression of COL1A1 and COL3A1 increased significantly in the anastomotic wounds from day 3 to day 5 in ZP1849-treated animals but not in vehicle-treated animals. This was also found for the soluble collagens in the anastomoses of the ZP1849-treated animals. Soluble collagen proteins represent newly synthesized collagen triple helices [26]. Although COL1A1 or COL3A1 mRNA levels or soluble fibrillar collagens were not significantly higher in ZP1849-treated animals compared with vehicle, this does not rule out that ZP1849 also influenced other aspects of collagen biology such as the ratio of collagen types, orientation, and/or cross-linking of the collagen molecules.

COX-2 is important for anastomotic wound healing, and COX-2-deficient mice show increased rates of AL [27]. This effect seems to be due to impaired angiogenesis rather than to decreased infiltration of neutrophils or macrophages [27]. In the present study, COX-2 expression increased postoperatively in the anastomosis and appeared to decrease from day 3 to day 5, while the expression of COX-1 was unaltered. This COX-1 and COX-2 mRNA profile is very similar to that reported by Reuter et al. in a rat model of colitis [28]. It should be emphasized that we used the COX-2 inhibitor meloxicam from postoperative day 1 for analgesia that may have downregulated gene expression and/or activity of COX-2 [29]. In another study in the same animal model, there was no impact of another COX-2 inhibitor on the anastomotic breaking strength or significant correlation between COX-2 levels and breaking strength [30]. Despite a potential inhibition of COX-2, ZP1849 treatment induced COX-2 gene expression. On the other hand, we found no effect of ZP1849 treatment on the anastomotic MPO levels. Taken together, these findings suggest that GLP-2 does not affect anastomotic wound healing through inflammatory pathways.

TGF-*β* is closely involved in collagen metabolism during early colonic anastomotic wound healing [31, 32]. We have previously shown a correlation between TGF-*β*1 and soluble collagen indicative of collagen synthesis in anastomotic healing on day 3 [19]. TGF-*β*1 levels were reduced with ZP1849 treatment on day 5, an observation also reported by Redstone et al. [13]. It is well-known that TGF-*β*1 induces the myofibroblast phenotype; therefore, ZP1849-treated animals may possess fewer myofibroblasts in the site of anastomoses [18, 33].

GLP-2 treatment has numerous beneficial effects in animal models of intestinal injury. These include improved intestinal barrier function, enhanced crypt cell proliferation, and inhibited apoptosis of epithelial cells [10, 34, 35]. The observed increased anastomotic extensibility with ZP1849 could reflect a more advanced mucosal regeneration. This may give a hint that the beneficial effect of ZP1849 was via stimulation of epithelialization more than modulation of the submucosal collagens.

Epithelialization is the net result of migration, proliferation, and apoptosis. MMP-7 is necessary for normal migration of the intestinal epithelium, and additional MMP-7 promoted the reconstitution of the denuded epithelium in vitro [36, 37]. ZP1849 treatment did not impact MMP-7 expression, which suggests that ZP1849 did not influence epithelial migration but rather cell proliferation. This was also indicated by the increased Ki67 expression with ZP1849. Ki67 labelling is predominantly found in the crypt epithelium in healing colonic anastomoses [18]. The mitogenic effect of GLP-2 on epithelial cells seems to be mediated by IGF-1 in a paracrine manner [38]. The reduced TGF-*β*1 levels would allow for increased epithelialization due to its antiproliferative effects on epithelial cells [39].

In summary, we have shown that the GLP-2 analogue ZP1849 improved the breaking strength of colonic anastomoses in a rat model, possibly via increased IGF-1 expression, suggesting that GLP-2 treatment is beneficial for colonic anastomotic healing.

## 5. Conclusions

The GLP-2 analogue ZP1849 increased anastomotic breaking strength, IGF-1 expression, and cell proliferation, which may be beneficial for colonic anastomotic wound healing.

## Figures and Tables

**Table 1:** Overview over primer sequences for mRNA analyses by RT-qPCR.

Target	Genbank accession no.	Sense	Antisense
RPLP0	NM_022402.2	CCAGAGGTGCTGGACATCACAGAG	TGGAGTGAGGCACTGAGGCAAC
GAPDH	NM_017008.4	CCATTCTTCCACCTTTGATGCT	TGTTGCTGTAGCCATATTCATTGT
GLP-2R	NM_021848.1	GTGGCCTTGCAGTATGGCTTTG	TAAGAAGCGGCCCCATGACTTT
Ki67	NM_001271366.1	TCCAGCTGGCCTAAAGAAAATCATCAA	TGAAGTCCTGCCTGATCTTCGTCT
IGF-1	NM_178866.4	GGAGGCTGGAGATGTACTGTGCT	TGTGTTCTTCAAGTGTACTTCCTTCTG
COL1A1	NM_053304.1	ATCAGCCCAAACCCCAAGGAGA	CGCAGGAAGGTCAGCTGGATAG
COL3A1	NM_032085.1	TGATGGGATCCAATGAGGGAGA	GAGTCTCATGGCCTTGCGTGTTT
COX-1	NM_017043.4	TTGGCCTGAAGCCTTACACTTCTTT	TCAGCGGCCATCTCCTTCTCTC
COX-2	NM_017232.3	CCTTGAACACGGACTTGCTCACTT	AATGGAGGCCTTTGCCACTGCT
MMP-7	NM_012864.2	AAAGGACGACATTGCAGGCATC	GAAGGGCGTTTGCTCATTCCA

**Table 2:** MPO and TGF-*β*1 proteins in colonic anastomotic wounds on days 3 and 5.

	*n*	Day 3	Day 5
MPO (ng/mg protein)			
Vehicle	16	14.7 ± 2.9	12.0 ± 3.1
ZP1849	16	14.2 ± 2.1	11.9 ± 2.2
TGF-*β*1 (pg/mg protein)^a^			
Vehicle	16	247 ± 45	292 ± 88
ZP1849	16	230 ± 47	200 ± 62^∗∗∗^

Mean ± SD. MPO: time, *P* < 0.001. TGF-*β*1: treatment × time, *P* = 0.018. ^∗∗∗^*P* < 0.001 compared with the vehicle group. ^a^Samples were activated with 1 N HCl.

## Data Availability

The data used to support the findings of this study are available from the corresponding author upon request.

## Supplementary Materials

Supplementary Materials Comparison of two biomechanical methods as surrogate metrics of anastomotic wound healing in the left colon of male rats.
