# Supplementary material for: Glucagon-Like Peptide-2 Analogue ZP1849 Augments Colonic Anastomotic Wound Healing
Source: Gastroenterol Res Pract. 2020 Oct 9;2020:8460508. doi: 10.1155/2020/8460508 (PMC7568816; doi:10.1155/2020/8460508)
Supplement: Supplementary Materials — Comparison of two biomechanical methods as surrogate metrics of anastomotic wound healing in the left colon of male rats. [file 8460508.f1.docx]

# Supplementary Material

# Comparison of Two Biomechanical Methods as Surrogate Metrics of Anastomotic Wound Healing in the Left Colon of Male Rats

The most common biomechanical metrics for anastomotic wound healing are anastomotic bursting pressure (ABP) and anastomotic breaking strength (ABS). ABP reflects the integrity of the anastomosis by its resistance to increased intraluminal pressure, whereas ABS reflects the resistance to increased longitudinal load required to break the entire anastomotic line.

ABP and ABS measures have their advantages and disadvantages. ABS increases in parallel to the deposition of new collagen in the anastomosis up to day 7 and thus mirrors the physiological processes of wound repair [1, 2]. The major problem encountered with ABP measurements at this stage of healing is disruption of the anastomosis outside the anastomotic line which invalidates the measurements [3].

The objective of this study was to compare ABP and ABS measurements in early anastomotic healing in the left colon of male rats. In addition, the feasibility and reliability of performing ABP and ABS on the same anastomotic specimen 3 and 5 days after the construction of the anastomoses was investigated.

## Materials and Methods

End-to-end single layer anastomoses were constructed using 8 interrupted, inverted polypropylene monofilament 6/0 sutures (Prolene®) placed 2 mm from the resection margins in anaesthetized 80 male Wistar (Charles River Laboratories) rats (262-415 g). On postoperative days 3 and 5, 20 rats were randomized to group A—where ABP was measured before ABS on the same anastomosis specimen—or to group B—where ABS alone was measured. The study was carried out in accordance with guidelines from The Ministry of Environment and Food of Denmark (2016-15-0201-01037). The ARRIVE guidelines were followed.

**ABP measurements**

ABP was measured using an infusion pump (SP220, KD Scientific Inc., Holliston, MA, USA) coupled to a calibration device with an integrated pressure generator (KAL84, Kastrup-Walther GmbH, Kirchzarten, Germany). One end of the colonic segment was closed with a 2.0 silk thread and a surgical vessel clamp. From the other end of the segment, a soft thin silicone tube with a small plug was introduced at 1 cm, fixed with a 2.0 silk thread and vessel clamp and attached to the bursting pressure device (Figure S1). The colonic segment was immersed in saline (20-22°C) and inflated with air (2.5 mL/minute). ABP was defined as the maximum pressure in mm Hg just prior to an abrupt drop in pressure or when small bubbles appeared.


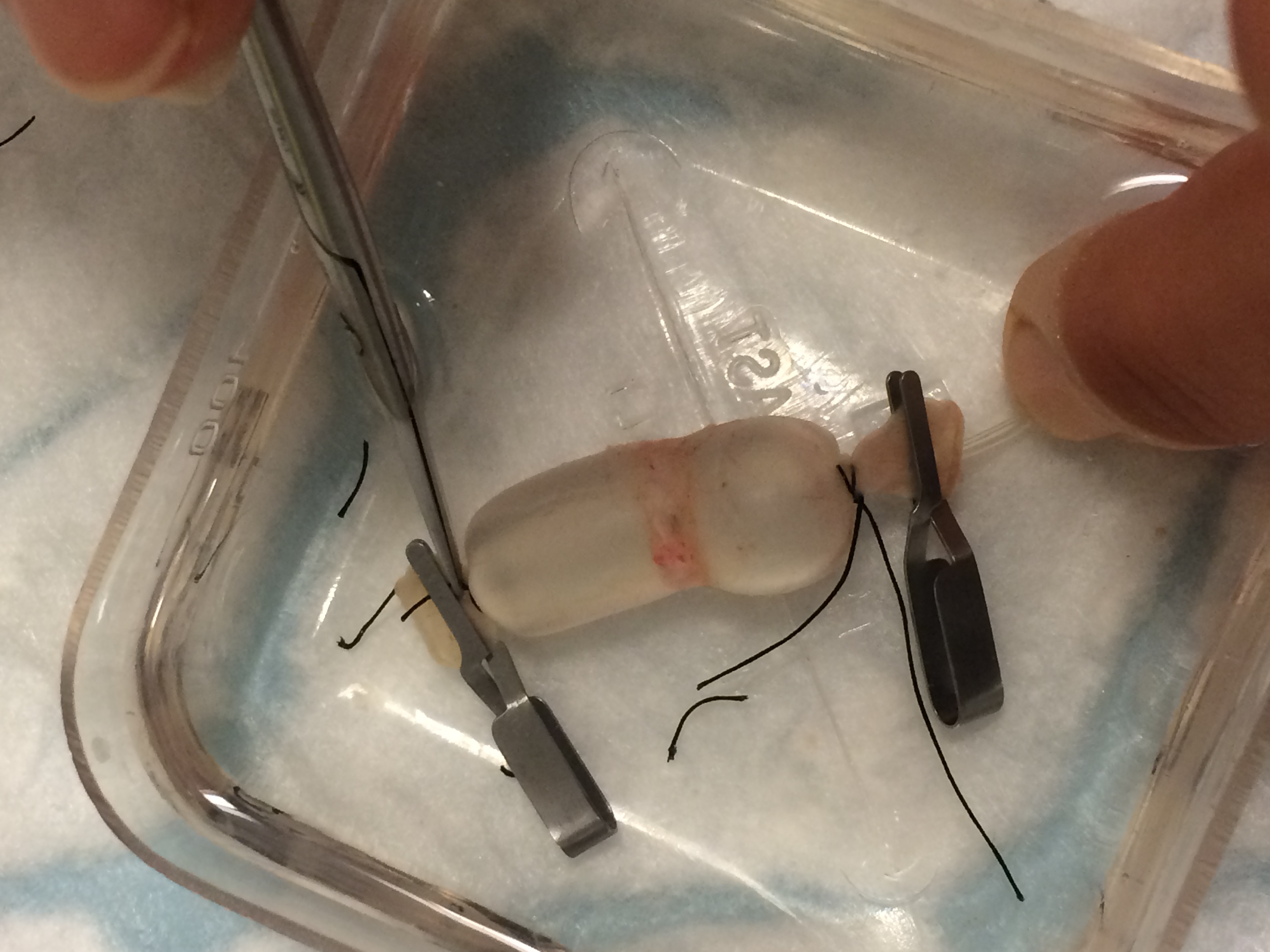


Figure S1: The ABP test. Inflated colonic segment including the anastomosis. Note the narrowing in the anastomotic line.

**ABS measurements**

The colonic segment was mounted on a vertical materials testing machine (LF Plus, Lloyd Instruments, Bognor Regis, UK), with a 10-mm gap between the clamps (Figure S2a). The segment was stretched at a constant speed (10 mm/minute) until anastomotic rupture. Load (force) and deformation (elongation) values were recorded from the load-deformation curve generated by Nexygen software (Lloyd Instruments). The ABS (ultimate load) in N was defined as the maximum force at rupture of the anastomosis. Extensibility (%) and energy absorption (N ⋅ mm) are defined in Figure S2b.

(a) (b)


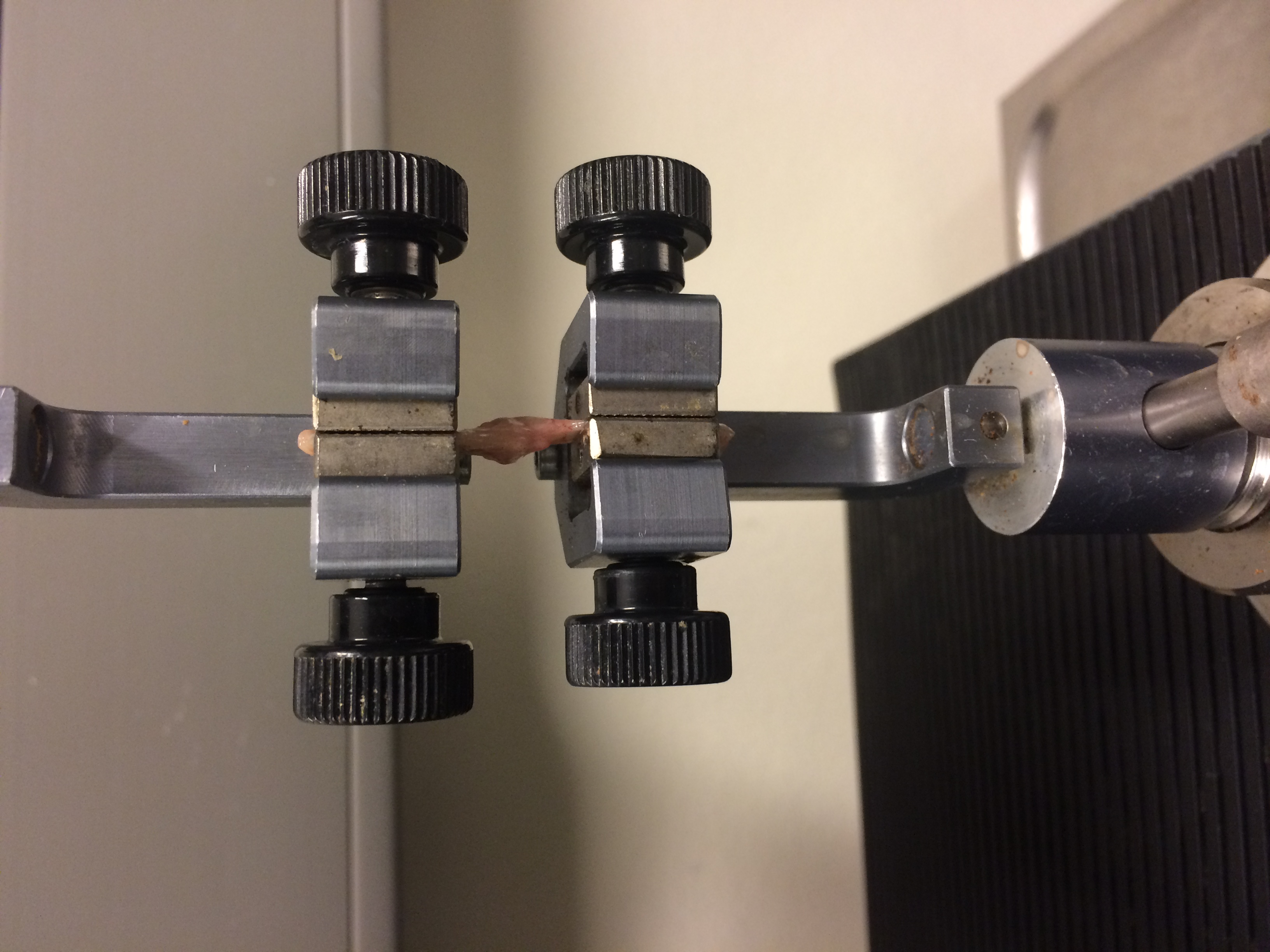

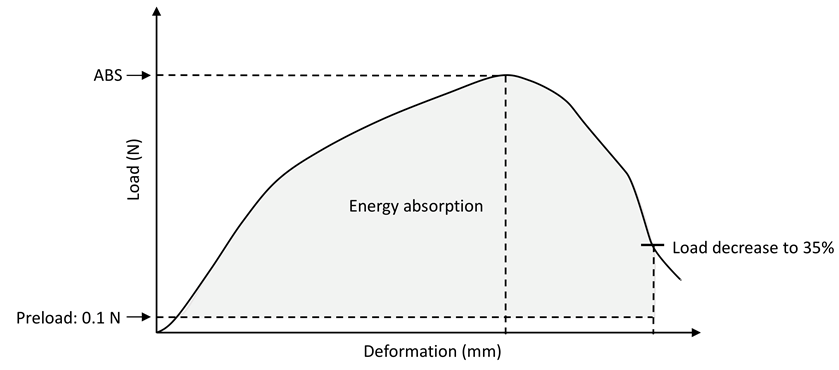


Figure S2: The uniaxial ABS test. (a) The colonic segment with the anastomosis is clamped in the testing machine. (b) Extensibility (%) was defined as the length the specimen could be elongated at ultimate load (ABS) divided by its initial reference length. The reference length was defined as the sum of the gap between the clamps (10 mm) and the elongation that occurred until the preload value of 0.1 N was obtained [3]. The energy absorption (N ⋅ mm) of the anastomosis was calculated as the area under the load-deformation curve (grey) from the starting point to the breaking point defined by 35% of ABS.

## Results

On day 3, all the 14 anastomoses (100%) burst in the anastomotic line. On day 5, 4 of the 17 anastomoses ruptured outside the anastomotic line. The values of these were 147, 148, 180 and 180 mmHg. The ABP of the remaining 13 (76%) anastomoses varied from 21.3 to 189 mm Hg. All anastomoses (100%) broke in the anastomotic line when measuring ABS days 3 and 5. The variability (CV) in the ABP measurements increased (P < 0.001) from day 3 to day 5, whereas the variability of the ABS measurements did not change significantly from days 3 to 5 (Table S1).

Table S1: The effect of day of assessment on ABP (group A) and ABS (group B).

|  | n | Outcome | Variability (CV) | *P* |
| --- | --- | --- | --- | --- |
| ABP (mm Hg) |  |  |  |  |
| Day 3 | 14 | 55.0 (32.3-64.5)^a^ | 45.8% | < 0.001^b^ |
| Day 5 | 13 | 91.4 (40.4-145)^a^ | 67.0% |  |
| *P* |  | 0.026^c^ |  |  |
| ABS (N)* |  |  |  |  |
| Day 3 | 20 | 1.34 ± 0.37^d^ | 28.0% | 0.666^e^ |
| Day 5 | 19 | 1.74 ± 0.34^d^ | 19.3% |  |
| *P* |  | 0.002^f^ |  |  |
| ABP, anastomotic bursting pressure. ABS, anastomotic breaking strength. CV, coefficient of variation. ^a^Median (interquartile range). ^b^Nonparametric Levene’s test. ^c^Mann-Whitney test. ^d^Mean ± SD. ^e^Levene’s test. ^f^Unpaired t-test. *The anastomoses had not been subjected to the ABP test before ABS was measured. | | | | |

The ABS did not differ between group A and group B on day 3 (*P* = 0.874) or day 5 (*P* = 0.789). The anastomotic extensibility was lower (*P* = 0.029) in group A versus group B on day 5 (Table S2).

Table S2: The effect of performing ABP measurements before ABS (group A) or not (group B) on biomechanical outcomes.

|  | n | ABS (N) | Extensibility (%) | Energy absorption (N ⋅ mm) |
| --- | --- | --- | --- | --- |
| Day 3 |  |  |  |  |
| Group A | 14 | 1.32 ± 0.38 | 89.0 ± 24.3 | 10.0 ± 4.5 |
| Group B | 20 | 1.34 ± 0.37 | 112 ± 41.4 | 10.4 ± 6.3 |
| *P*^a^ |  | 0.874 | 0.245 | 0.904 |
| Day 5 |  |  |  |  |
| Group A | 17 | 1.77 ± 0.41 | 88.4 ± 45.1 | 14.4 ± 6.5 |
| Group B | 19 | 1.74 ± 0.34 | 119 ± 39.9 | 17.7 ± 5.1 |
| *P*^a^ |  | 0.789 | 0.029 | 0.062 |
| Mean ± SD. ABP, anastomotic bursting pressure. ABS, anastomotic breaking strength. ^a^Unpaired t-test. | | | | |

ABP did not correlate with ABS on either day 3 or day 5 (Figure S3) indicating that ABS and ABP reflect different biomechanical phenomena during anastomotic wound healing in the colon.


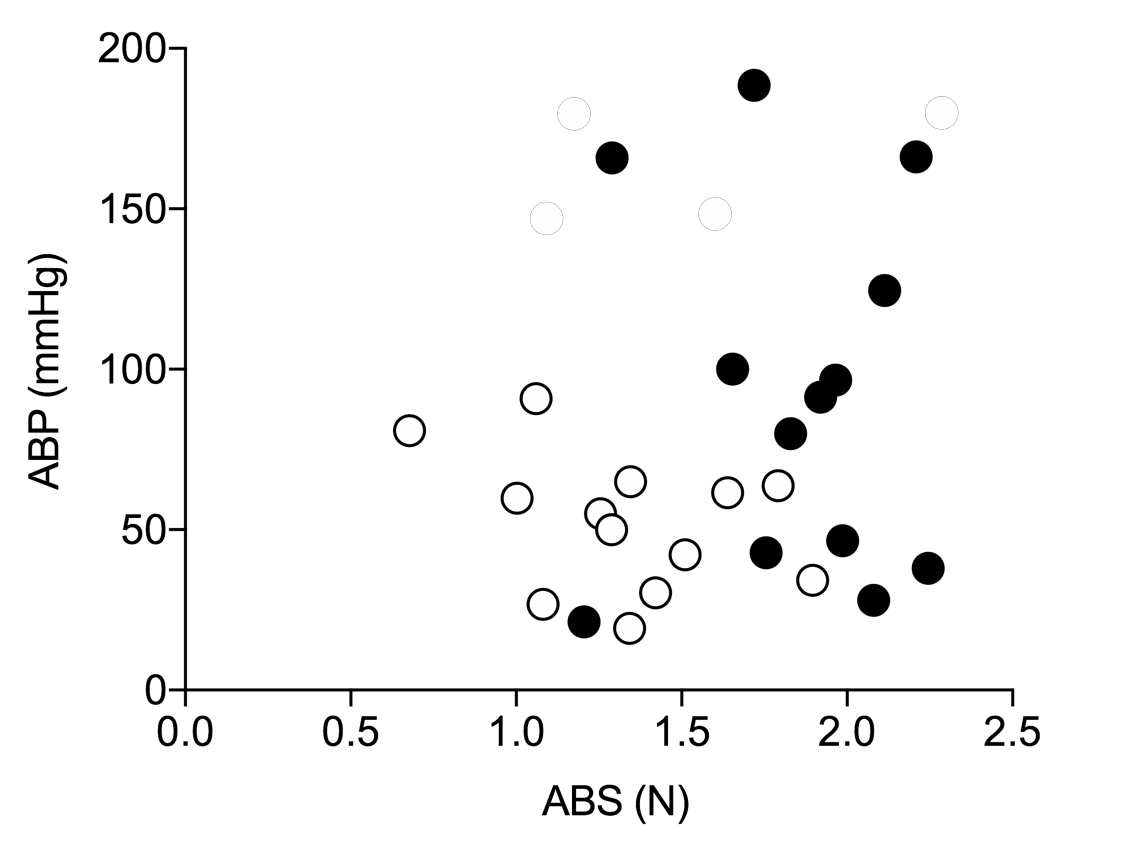


Figure S3: ABP plotted against ABS on postoperative day 3 (rs = -0.285, *P* = 0.425) and postoperative day 5 (rs = -0.082, *P* = 0.771). Day 3, open symbols. Day 5, closed symbols. ABP, anastomotic bursting pressure. ABS, anastomotic breaking strength.

## Conclusions

There is no consensus on the optimal use of biomechanical measures in colonic anastomotic wound healing research. ABP is less suitable due to increased rupture outside the anastomotic line and increased variability compared with ABS measurement, and in our hands, ABS is the preferable method to evaluate the healing of colonic anastomoses.

## Data Availability

The data used to support the findings of this study are available from the corresponding author upon request.

## References

[1] Jiborn H, Ahonen J, Zederfeldt B (1978) Healing of experimental colonic anastomoses. II. Breaking strength of the colon after left colon resection and anastomosis. Am J Surg 136:595-599.

[2] Ikeuchi D, Onodera H, Aung T, et al. (1999) Correlation of tensile strength with bursting pressure in the evaluation of intestinal anastomosis. Dig Surg 16:478-485.

[3] Christensen H, Flyvbjerg A, Ørskov H, Laurberg S (1993) Effect of growth hormone on the inflammatory activity of experimental colitis in rats. Scand J Gastroenterol 28:503-511.
